# Clinical skills development for healthcare practitioners working with patients with persistent physical symptoms (PPS) in healthcare settings: a systematic review and narrative synthesis

**DOI:** 10.1186/s12909-024-05306-4

**Published:** 2024-03-22

**Authors:** Stacie Thursby, Lorelle Dismore, Katherine Swainston

**Affiliations:** 1 Northumbria Healthcare NHS Foundation Trust, North Tyneside General Hospital, North Shields, NE29 8NH United Kingdom; 2 Faculty of Medical Sciences, School of Psychology, Newcastle University, 4 Floor Dame Margaret Barbour Building, Wallace Street, Newcastle Upon Tyne, NE1 7RU England

**Keywords:** Persistent physical symptoms, Functional somatic symptoms, Medically unexplained symptoms, Clinical skills, Healthcare practitioner training, Behaviour change, Systematic review

## Abstract

**Background:**

The complexity and uncertainty around Persistent Physical Symptoms (PPS) make it difficult to diagnose and treat, particularly under time-constrained consultations and limited knowledge. Brief interventions that can be utilised in day-to-day practice are necessary to improve ways of managing PPS. This review aimed to establish (i) what training primary and secondary healthcare practitioners have undertaken to develop their clinical skills when working with PPS, (ii) what training techniques or theoretical models have been used within these interventions, and (iii) how effective was the training.

**Method:**

A systematic literature search was undertaken on eight databases to identify professional development interventions for healthcare practitioners working with PPS, were of any study design, and at a minimum were single measure studies (i.e., training outcome alone). Studies were assessed using the Mixed Methods Appraisal Tool (MMAT) and narratively synthesised.

**Results:**

Despite high methodological heterogeneity across the six included studies, they all aimed to improve healthcare practitioners’ communication skills through educational (theory, awareness, attitudes, assessment, treatment, and management of PPS) and experiential (role play) learning.

**Conclusions:**

The review findings demonstrate that developing healthcare practitioners’ communicative behaviours led to increased confidence and self-efficacy when working with PPS, which facilitated improved consultations and improvements on some patient outcomes. Barriers to the uptake of training programmes and implementation into daily clinical practice are discussed, including the need for PPS to be formally implemented into undergraduate teaching and post-qualification continuous professional development.

**Trial registration:**

This review was registered at PROSPERO [CRD42022315631] prior to the review starting.

## Background

An increasing number of patients across healthcare settings are presenting with physical complaints which, after medical examination, sufficient somatic explanation cannot be identified [1]. These complaints are referred to as *persistent physical symptoms* (PPS) and are also known as *medically unexplained symptoms* (MUS) or *functional somatic symptoms* (FSS). PPS represent a broad and heterogeneous spectrum of symptoms e.g., pain, headaches, dizziness, and conditions such as fibromyalgia, chronic fatigue, irritable bowel syndrome [2, 3]. Excessive and frequent healthcare utilisation of patients with PPS [4–6] puts this group of patients among the highest costing group within the National Health Service (NHS) [7, 8], with further costs to the economy including high rates of sickness absence [2]. The cost of PPS to patients includes functional impairment, reduced quality of life, and psychological and emotional distress [7, 9]. The complexity and uncertainty around PPS, however, make it difficult to diagnose and treat, and ways of better managing symptoms and conditions are urgently required [10].

The lack of understanding of PPS often leads to patients undergoing inordinate levels of symptomatic investigation and medical intervention in biomedically-focused healthcare systems, increasing the risk of iatrogenic harm [11, 12]. Adjunct ways of working with patients is through psychological intervention. A recent meta-analysis identified that various psychological therapies are effective for managing PPS, including reducing somatic symptoms [13]. Moving away from the dualistic approach in healthcare and towards a more holistic approach to assess, treat, and manage patients is recommended e.g., NICE guidelines [14, 15] for PPS-related conditions. Murray et al. [16] draws from the etiopathogenetic model from non-specific, functional, and somatic complaints, enunciating that PPS symptoms can be triggered and perpetuated by psychological, biological, and sociocultural factors, that also play a predisposing role. Therefore, addressing these factors holistically is likely to improve health outcomes for patients thus improving clinical outcomes, reducing repeat consultations and medical costs.

Whilst psychotherapies can be an effective alternative to medical treatment for the management of PPS, brief interventions are necessary to ensure better utility in day-to-day practice [13, 17]. PPS identified in general practice and patients receiving support to cope better with symptoms, rather than seeking a cure, can improve their quality of life and prevent symptoms from becoming chronic and disabling [7]. Simple techniques include opening the conversation to identify psychosocial issues and addressing adverse health behaviours that can exacerbate symptoms [18]. Consultation studies report that ineffective communication prevents general practitioners (GP) from exploring patients’ ideas and expectations of their symptoms in-depth [19] and that clinicians will often ignore psychosocial cues [9, 20]. However, when clinicians do attempt to discuss non-somatic contributing factors to PPS, patients feel misunderstood or offended due to their lay beliefs that physical disease is the cause of their symptoms [21, 22].

The discrepancies between clinicians’ and patients’ agendas form difficult barriers in PPS consultations. Clinicians often feel unequipped to find agreement and understanding of symptoms [18, 22], consequently providing little reassurance for their patients [9]. Despite patients often seeking medical intervention, they have also reported seeking non-pharmacological solutions including emotional support and plausible explanations for their symptoms [23, 24]. It is important that patient satisfaction during consultations is improved as this will encourage active participation in their own healthcare, which is crucial for the process of adaptation to and recovery from illness [21].

Various training programmes have been developed and delivered aiming to improve healthcare practitioners’ clinical skills and patient outcomes, however, they often differ in content, technique, underpinning theoretical models, and measures used to assess the training. A systematic review is required to identify, critically appraise, and assess the potential effectiveness of existing training intervention programmes to identify limitations and develop guidance for those working with PPS.

### The Present Study

The aim of this review is to establish (i) what training primary and secondary healthcare practitioners have undertaken to develop their clinical skills when working with PPS, (ii) what training techniques or theoretical models have been used within these interventions, and (iii) how effective was the training.

## Method

The present review adhered to the methodological processes outlined in our protocol as registered on PROSPERO [CRD42022315631] and complies with the Preferred Reporting Items for Systematic Reviews and Meta-analyses (PRISMA) guidelines [25].

### Search Strategy and Sourcing Information

An extensive literature search was performed on eight electronic databases in June 2022 and a re-run was undertaken in March 2023 to ensure any new literature was captured: CINAHL, EMBASE, MEDLINE, Psychology and Behavioural Science Collection, Nursing and Allied Health Source, PsychINFO, Scopus, and The Allied and Complementary MEDicine Database. The reference lists of included articles were subsequently searched to identify any additional studies. Search terms were defined by the review question and are specific to professional development interventions delivered to healthcare practitioners to improve service delivery and outcomes for patients with PPS. There were no restrictions on publication date, however, only studies written in English or that could be translated to English were included in the review.

Population search terms were carefully chosen based on current and historical definitions to identify ongoing physical complaints with no obvious pathology. PPS is currently used as the preferred term, with MUS previously used extensively by clinicians and researchers. However, the term MUS prioritises medical explanation and reinforces mind–body dualism, whereas PPS considers how the intricate process between biological, psychological, and social factors influence the development of physical complaints [26]. Marks and Hunter [26] found that up to one-fifth of their sample preferred the terms PPS (20%) and Functional Symptoms (17%), with 15% endorsing MUS. Therefore, PPS, MUS and FSS were included in the search strategy.

An example of the search strategy is illustrated in Table 1, and full searches are tabulated in Table 4 in Appendix.

**Table 1:** Search Strategy: Search Terms [PICO: Population, Intervention, Comparison, Outcome

Population	Intervention	Comparison	Outcome
Persistent Physical Symptoms Medically Unexplained Symptoms Functional Somatic Symptoms	Training Learning Development	One intervention compared against another OR A single measure i.e., training outcome alone	Service delivery Healthcare provision Quality of care Clinical skills Physician–patient relationship

### Study Eligibility and Selection Criteria

Eligibility criteria were qualitative or quantitative studies in any peer-reviewed journals that encompassed a training intervention meeting the aims of the review. The following inclusion criteria were used: (i) a clearly defined training intervention was reported, (ii) the sample being trained were healthcare practitioners in primary or secondary care, (iii) all patients included in the study sample experienced persistent physical symptoms, (iv) any study design e.g., randomised control trial, pre-post studies, intervention only.

### Screening

Following removal of duplicate articles, the remaining titles and abstracts were screened independently by two reviewers (ST, LD) in Rayyan software. All articles recoded as *‘include’* or *‘maybe’* were reviewed at full text, which were screened by two reviewers (ST, LD). Conflicts of opinion were resolved via discussion with the third reviewer (KS).

### Data Extraction

Two reviewers (ST, LD) extracted data using an agreed data extraction format. The extraction tool recorded: study sample e.g., age, profession; study details e.g., design, quality assessment; mode of healthcare e.g., primary care, secondary care; intervention e.g., education, experiential; outcome e.g., communication; and study findings e.g., effectiveness of the intervention and areas for service improvement.

### Quality Assessment and Risk of Bias

The quality of included studies was assessed using the Mixed Methods Assessment Tool (MMAT) for systematic multi-method reviews [27]. Two authors (ST, LD) independently quality appraised the included studies with any disagreements resolved via discussion with the third author (KS).

### Data Synthesis

Due to the methodological heterogeneity of included studies, a meta-analysis was not appropriate therefore a narrative synthesis was performed guided by the Economic and Social Research Council Methods Programme framework: *Guidance on the Conduct of Narrative Synthesis for Systematic Reviews* [28]. The results of the included studies were inputted verbatim into NVivo software for sorting, coding, and synthesising, which facilitated the tabulation of results to support the identification of patterns across studies. Subsequent pooling of emerging commonalities into smaller groups enabled the processes of description and analysis across groups, leading to the synthesising of data. Intervention effects can then be explored by means of subgroup analysis where methodological diversity is present [28], which in the present review, was examined via mode of healthcare.

## Results

**Fig. 1:**
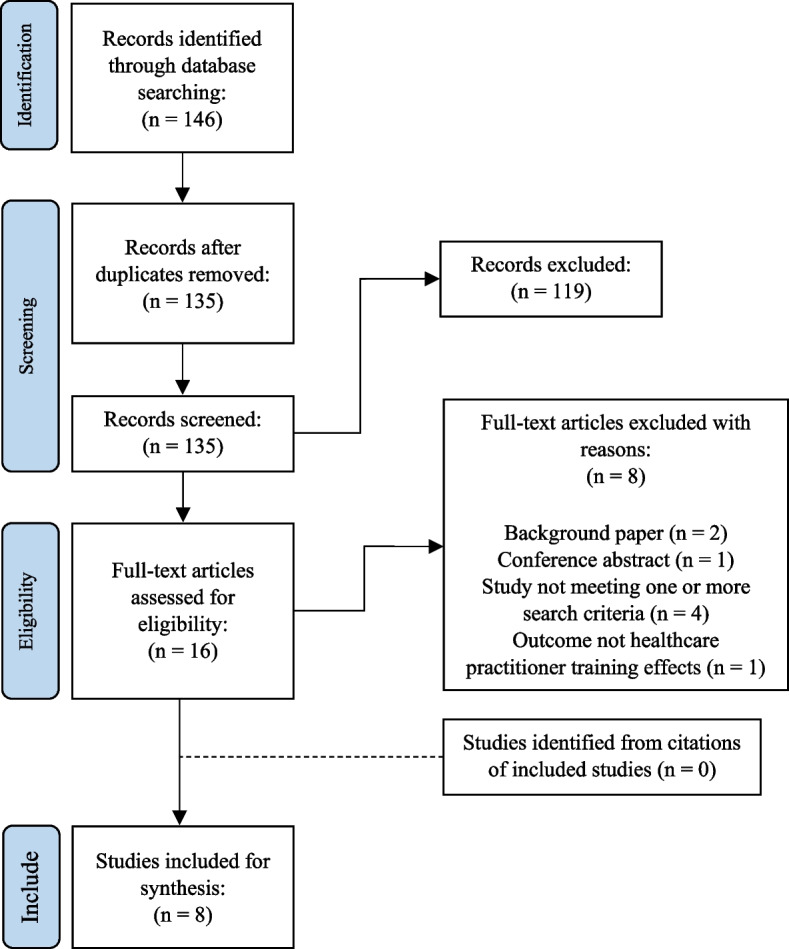
PRISMA Search Results

The screening process and search results are shown in Fig. 1. A total of 146 results were returned after duplicates were removed, 16 studies were assessed at full text and 8 papers were suitable for inclusion in the review [20–22, 29–33]. It should be noted that six studies in total were conducted, however different aspects of Morriss and colleagues’ study were analysed and written up in multiple papers at different time points. The three papers focused on feasibility of the intervention [31], a full trial of the intervention [32], and observations of psychosocial chatter during consultations [20]. Demographics of included studies are summarised in Table 2.

**Table 2:** Study demographics of included studies

Reference		Country	Level of Care	Participants		
				HCPs	Profession	Patients
1	Abrahamsen et al. (2022) [29]	Norway	Primary	IG (*n* = 8) CG (*n* = 0)	GPs	*n* = 0
2	Frostholm et al. (2005) [21]	Denmark	Primary	IG (*n* = 18) CG (n = 20)	GPs	IG (n = 778) CG (n = 734)
3	Houwen et al. (2022) [30]	The Netherlands	Primary	IG (*n* = 31) CG (*n* = 0)	GP Residents	*n* = 0
4	Morriss et al. (2006, 2007, 2010) [20, 31, 32]	UK	Primary	IG (*n* = 35) CG (*n* = 40)	GPs (*n* = 74) Nurse (*n* = 1)	IG (*n* = 66) CG (*n* = 75)
5	Schaefert et al. (2013) [33]	Germany	Primary	IG (*n* = 18) CG (*n* = 17)	GPs	IG (*n* = 170) CG (*n* = 153)
6	Weiland et al. (2015) [22]	The Netherlands	Secondary	IG (*n* = 62) CG (*n* = 61)	Residents (*n* = 74) Specialists (*n* = 49)	IG (*n* = 229) CG (*n* = 220)

*Abbreviations:* *HCPs* (Healthcare Practitioners), *IG* (Intervention Group), *CG* (Control Group), *GP* (General Practitioner)

### Study Characteristics

Study characteristics of included studies are summarised in Table 3. Five studies were randomised control trials [20–22, 32, 33], one study was cross-sectional [31], one study was qualitative [29], and one study was mixed methods [30]. Studies were carried out in Norway, Denmark, the Netherlands, United Kingdom (UK) and Germany, with one study taking place in secondary care [22] and the remaining studies in primary care. All training programmes incorporated educational and practical elements, and were mixed in delivery i.e., three studies were delivered via blended learning which offers an educational approach that integrates online and face-to-face learning [21, 29, 30] and the remaining three were in-person only.

**Table 3:** Study characteristics of included studies

	**Reference**	**Design**	**Intervention** **(features)**	**Mode of Delivery**	**Outcomes**	**Evaluative factors**
1	Abrahamsen et al. (2022) [29]	Qualitative	The ICIT (Individual Challenge Inventory Tool): A conversational tool with elements of CBT Underpinned by Bandura’s Social Learning Theory	Blended learning 18 h over 4 days in an 8-week period Video, theory, role play, video consultation reviews (healthcare practitioners reviewing their own consultations)	Primary HCPs: Focus group feedback. Analysed using Manual Analysis Secondary n/a	The conversation tool helped structure consultation; patients achieved sense of control (viewed as an important self-help tool); useful to help patients reflect on positives, rather than limitations
2	Frostholm et al. (2005) [21]	RCT	The Extended Reattribution Model (TERM): A multifaceted educational programme for the assessment, treatment, and management of PPS	Blended learning 16 h over 2 days + 3–4 evening courses + one 2-h booster meetings after 6 months Theoretical presentations covering PPS conceptualisations, etiology and epidemiology, patients’ illness beliefs, and iatrogenic factors Skills training via video supervision	Primary PSCQ-7 Secondary HCP questionnaire IPQ SCL-8 WI-7 SCL-90 SCL-SOM CAGE-4	n/a
3	Houwen et al. (2022) [30]	Mixed Methods	Intervention Mapping Framework: A systemically developed communication training programme Steps 1: Needs assessment; 2: Formulate change objectives; 3: Methods and applications; 4: Develop programme; 5: Implementation; 6: Evaluation	Blended learning 6 h over 2 days + 7 × 45–60-min online modules Online modules Awareness, attitudes, knowledge, assessment, and treatment of PPS, psychological treatment (optional), collaboration with other HCPs Face-to-face Role play focusing on attitude, exploration and shared understanding of PPS including empathy and psychosocial issues, explanations and taking control	Primary Quantitative: SE-12 Qualitative: Interviews Secondary n/a	HCPs appreciative of the blended learning delivery of the programme Online course good theoretical preparation for in-person training days HCPs reported the e-learning to be extensive and time consuming
4	Morriss et al. (2006, 2007, 2010) [20, 31, 32]	Cross-sectional	The Extended Reattribution Model: A communication programme to provide a simple three stage psychological explanation for PPS through negotiation Main aspects included: symptom, psychosocial issue, and identifying physical or temporal mechanism that links symptoms and psychosocial issue(s)	In-person only Three 2-h sessions Was four in previous trial but reduced to 3 as there was a 51% drop-out rate Overall, the training included: Skills, attitude, and knowledge of PPS; Improving ability to recognise patient’s problems (e.g., worry of symptoms, emotions); Explain how problem(s) are linked to symptoms; Patient-centred approach; Order of fewer referrals, investigations, and drugs; Increased active treatment of mental disorder Role play to practice skills	2006 HCP: 1 feasibility questionnaire 2007, 2010 HCPs: 2 × Assessed audio transcriptions of GP/patient consultations Transcripts scored on a 5-point Likert scale measured by how consistent GPs’ communication was in line with reattribution Transcripts were scored on a 5-point Likert scale observing the frequency of psychosocial chatter. Scoring was guided by the Liverpool Clinical Interaction Analysis Scheme (LCIAS)	2006 New learning achievements following training: 48% better or alternative ways of making the link 22% provision of structure to consultations 18% more confidence to openly discuss PPS with patients 2007, 2010 n/a
5	Schaefert et al. (2013) [33]	RCT	Specific Collaborative Group Intervention: A patient group intervention focused on an interpersonal approach with psychodynamic factors All GPs were training in the diagnosis and management of PPS, then split off into the IG or CG. Difference between groups: In the IG, the GP was working in collaboration with a psychodynamic specialist; specialist input was minimal	In-person only 15.5 h over 2 evenings and 1 day Focus: Attitudes, treatment, knowledge, and skills around PPS; an interpersonal perspective and the use of patient-centred communication to build a sustainable working alliance; illness beliefs, the biopsychosocial model, using treatment tools, and supporting the use of active coping skills were also covered Group discussions and role play were enacted based on patient-centred communication	Primary Patients: SF-36 (specifically the Physical Composite Score at 12 months) Secondary SF-36 (specifically the Mental Composite Score at 12 months) Clinical symptoms Psychosocial distress Healthcare utilisation PHQ-15 PHQ-9 PHQ anxiety module PHQ panic module PHQ psychosocial stress measure WI-7 Use of antidepressants Patient-reported visits to medical specialist Medical Assessment Questionnaire	n/a
6	Weiland et al. (2015) [22]	RCT	An evidence-based communication programme using techniques from CBT to improve HCP interviewing, information-giving, and planning skills in PPS consultations	In-person only 14 h over 4 sessions in a 4–6-week period Educational, practical, and video consultation reviews (healthcare practitioners reviewing their own consultations) Sessions covered providing plausible explanations for PPS including making a link between interrelated factors that reinforced condition i.e., symptoms, cognitions, emotions, behaviour, and social factors; and avoiding unnecessary diagnostic testing Skills practice focused on patient centred communication to explore biopsychosocial factors, reassuring patients, and managing expectations	HCPs: Assessed audio transcriptions of GP/patient consultations Transcripts were scored on a 5-point Likert scale observing the application of PPS-focused communication skills. Scoring was guided by an adapted version of the Four Habit Coding Scheme	n/a

*Abbreviations*: *HCPs* (Healthcare Practitioners), *PSCQ-7* (Patient Satisfaction Consultation Questionnaire), *SCL-8* (Symptom Checklist), *WI-7* (Whitley Index – Health Anxiety), *SCL-SOM* (SCL-90 Somatisation Subscale), *CAGE-4* (Cutting, Annoyance, Guilty, Eye-opener), *SE-12* (Self-efficacy), *PSQ* (Patient Satisfaction Questionnaire), *EQ-5D* (European Quality of Life 5 Dimension), *QoL* (Quality of Life), *SF-36* (Short Form – Health Related Quality of Life), *PHQ* (Patient Health Questionnaire)

All training programmes aimed to improve healthcare practitioner communication skills, and programmes were developed based on the following conceptual models and frameworks: principles taken from Cognitive Behavioural Therapy (CBT) [22, 29], Abrahamsen and colleagues [29] specifically developed The Individual Challenge Inventory Tool (ICIT), a conversational tool underpinned by Bandura’s Social Learning Theory; The Extended Reattribution Model (TERM) [20, 21, 31, 32]; Intervention Mapping Framework [30]; and a Specific Collaborative Group Intervention (speciAL) focused on an interpersonal approach whilst integrating psychodynamic factors [33]. Two studies drew on the biopsychosocial model to inform the delivery of a holistic approach within consultations [22, 33].

### Quality Assessment of Included Studies

The studies demonstrated robust methodology and are considered strong overall in accordance with the MMAT scoring system, though some minor limitations should be acknowledged. Abrahamsen et al.’s [29] qualitative study reported on patient experiences, however, this was done through healthcare practitioners’ feedback of their interpretation and opinion of how patients experienced their consultations. A mixed methods design carried out by Howen et al. [30] reported very brief data and did not synthesise their results to corroborate findings. Drawbacks within two of the RCTs were either outcome assessors not being blinded to the intervention or uncertainty on whether they were blinded [21, 33].

### Narrative Synthesis

#### Study Features

All included studies featured educational and practical experiential learning. Educational aspects included covering theory relevant to PPS, assessment, treatment and management of PPS, and awareness and attitudes towards patients who present with PPS. Clinical skills development was done through role play which focused on interview techniques and information-giving i.e., providing plausible explanations for patients’ symptoms and creating a shared understanding through making the link between interrelated factors (symptoms and psychosocial stressors). In two studies, learning was also supplemented by healthcare practitioners reviewing their own videotaped consultations [22, 29].

#### Outcomes

A total of 36 quantitative outcomes and two qualitative outcomes were identified across the included studies, with various methodological designs utilised in each. Some studies observed the development of healthcare practitioner communication skills as their primary outcome, whilst others focused on patient outcomes. Healthcare practitioner quantitative outcomes were measured primarily through the scoring of doctor-patient communication using Likert scales or a coding framework [20, 22, 32], other measures included a single evaluative questionnaire [31], healthcare practitioner self-efficacy [30], and qualitative outcomes included focus groups and interviews [29, 30]. Patient primary outcomes include patient satisfaction [21] and physical components of quality of life [33].

Secondary outcomes varied widely with the main areas covered being somatic symptom severity, psychiatric factors including anxiety, depression, panic and alcohol misuse, quality of life particularly the mental health domain, and psychosocial distress. Healthcare utilisation was also observed i.e., number of appointments patients attended before and after healthcare practitioner training, patients medically assessed by healthcare practitioners, and the use of medication such as antidepressants [Table 3].

#### Effects of the training: Healthcare Practitioners

##### Application of techniques in clinical practice

A cross-sectional questionnaire observing the feasibility of the training identified that healthcare practitioners felt the techniques taught in their training programme were *useful* or *very useful* in their day-to-day practice [31]. This was supported by Houwen et al. [30], where it was reported in interviews that the training was helpful to improve communication with patients during consultations. Healthcare practitioners also reported increased confidence and self-efficacy to treat and manage patients who present with PPS. Further to this, confidence to openly discuss PPS had increased after taking part in the training programmes. Better or alternative ways of making a link between interrelated factors (i.e., between symptoms and psychosocial stressors) was reported to be a new learning achievement by almost half (48%) of healthcare practitioners who took part in the training [31].

##### Behaviour change: moving towards a biopsychosocial model of healthcare

Doctor-patient communication that was measured using audio transcripts was scored/coded to determine if they were using the techniques taught during their respective programmes [20, 22, 32]. Scores indicated that healthcare practitioners who had undergone training were able to better communicate with patients during PPS consultations, in comparison to the control groups. Areas of improvement included the development of interviewing skills and information-giving abilities during consultations, leading to better engagement with patients.

Healthcare practitioners worked in a more person-centred way and conversations included them exploring patients’ health beliefs, discussing interrelated factors more frequently, and broadening the agenda such as identifying the impact of patients’ symptoms on emotion, social environment, and behaviour. These findings were also supported by feedback from healthcare practitioners interviewed after their training [30]. The most beneficial aspects of the training programme included developing skills to holistically explore symptoms, being more aware of the language they use, identifying their personal attitudes towards patients with PPS, creating a shared understanding with patients, and familiarity with different explanatory models.

##### Psychosocial chatter

Morriss and colleagues [20] ran further analyses to identify to what extent the training influenced psychosocial talk between healthcare practitioners and patients. Training substantially increased healthcare practitioners prompting of patients for psychosocial information concerning their symptoms (85%); despite psychosocial disclosures from patients in the intervention group increasing by approximately 50%, healthcare practitioners did not investigate further when a new disclosure was made. In some cases, the training did increase healthcare practitioners’ provision of psychosocial explanations. Scores demonstrated that healthcare practitioners’ speech was inclined towards the appropriateness of somatic intervention (average utterances = 6) versus psychosocial explanation (average utterances = 2). However, healthcare practitioners advocating somatic intervention had reduced overall, with an increase in psychosocial explanation suggesting a shift towards holistic consultations.

#### Effects of the training: Patients

##### Patient satisfaction

Patients consulting trained healthcare practitioners reported higher levels of significant [21] and non-significant [32] satisfaction, with psychosocial issues playing a contributing role in patient satisfaction. These included feeling down, worried, problems within family, and personality. Predictors of patient satisfaction also included illness perception before consulting with their healthcare practitioner, particularly, uncertainty of what is wrong with them, their symptoms stimulating feelings of helplessness, high illness worry, and high levels of emotional distress [21].

In terms of improved clinical communication, patients who had consulted a trained healthcare practitioner felt they had a better understanding of their symptoms and endorsed an emotional response [32] or attributed psychosocial issues to explain their symptoms [20]. Some findings, however, did identify that the training was associated with increased negative physical and psychological outcomes in comparison to controls. Patients reported worse self-ratings of overall health, and whilst non-significant, more cases of anxiety, and beliefs that their illness may last longer, that they will experience more serious health consequences, or that they have less control of their symptoms [32].

##### Quality of Life

One study measured the physical component section of quality of life at 12 months post-intervention and found non-significant improvements in both the intervention and control groups [33]; at 12 months, however, the physical functioning domain was significant in the intervention group. Significant improvements in symptom severity were also found until 6 months, and whilst improvements persisted to 12 months, they were no longer significant. Schaefert and colleagues [33] measured the mental component of quality of life and the results showed significant improvements in both groups, with slightly larger improvements in the intervention group (55% versus 34%). Post-intervention scores showed a 4-point increase overall, which the authors identified as clinically significant as this is the threshold used previously to determine clinical change in patients with PPS in primary care. At 12 months, the vitality and emotional functioning domains of the mental component section were significant. Patients reported overall psychological improvements including lower levels of health anxiety and psychosocial distress. Contrary to this, Morriss et al. [32] measured depression and anxiety, and reported no effects on psychological wellbeing post-training.

##### Utilisation of healthcare resources

In terms of the utilisation of healthcare resources, Morriss et al. [32] reported that the TERM training had no effects on how often patients visited their healthcare practitioner or volume of medication consumed. Schaefert et al. [33], however, reported a reduction in the frequency of patient visits to their healthcare practitioners in the intervention (significant changes) and control (non-significant) groups of the speciAL training. Furthermore, patients’ use of antidepressants had reduced in both groups, with a greater decrease in the intervention arm however this only reached significant at 6 months.

##### Structuring the treatment and management of PPS

Finally, patient experiences in Schaefert et al.’s [33] study were reported by healthcare practitioners’ interpretation of the patient’s perception of the ICIT intervention, and feedback they received from patients in consultations. Healthcare practitioners felt that the activity plan incorporated into patient treatment acted as an important self-help tool for patients, facilitating them to develop a sense of control of their personal circumstances. Using a structured tool in consultations gave healthcare practitioners something specific to work on with patients. This method of consultation, healthcare practitioners felt, encouraged patients to reflect in a more positive way in terms of what they can achieve, rather than focusing on their limitations.

#### Evaluative Factors

##### Mode of delivery

Three studies delivered blended learning training programmes that were well received by healthcare practitioners [29, 30], who reported that the online materials provided good theoretical preparation for the in-person training days [30]. Weiland et al.’s [22] face-to-face training programme was evaluated using a single questionnaire, scoring an average of 2.79 on a 3-point Likert-scale. Healthcare practitioners reported the training to be *very useful* for daily practice, particularly the literature overview, skills development, and duration of the training.

##### Areas for Improvement

Healthcare practitioners interviewed in Houwen et al. [30] reported that the e-learning, which lasted up 7 h, was extensive and time consuming. They also stated that they had difficulty providing plausible explanations after the training and would have therefore benefited from focusing more on explaining explanatory models. Further to this, findings from Morriss et al. [31] identified that approximately one-fifth of healthcare practitioners would have liked more time to practice role play (18%), would have liked the opportunity to discuss difficult cases, and techniques (18%), and would have benefited from identifying a method of structure to their consultations (22%).

The narrative synthesis has summarised key findings from the included studies, a full breakdown of each included studies’ results can be found in Table 5 in the Appendix.

## Discussion

This review has reported and summarised existing professional development training to improve healthcare practitioners’ clinical skills when working with patients who present with PPS. Methodology across the six included studies drew from various theoretical models, techniques, and employed different outcomes measures however they all aimed to improve healthcare practitioners’ communication skills within patient consultations. Increasing healthcare practitioners’ knowledge around PPS and supporting their development of pragmatic interviewing skills and information-giving techniques was seen to improve patient and healthcare practitioner outcomes. Patient satisfaction improved and healthcare practitioners’ confidence and self-efficacy increased when working with PPS. Some results did not show improvements in patient outcomes; however, this was potentially due to healthcare practitioners not being able to practice the techniques taught within the consultation time they had with their patients during the study period [32].

### Pertinent issues in today’s practice

The included studies provide an evidence-base for effective training programmes to improve working with PPS. However, barriers to the uptake and implementation of such programmes have been identified, including negative attitudes towards patients presenting with PPS [34, 35]. Addressing healthcare practitioners’ attitudes towards patients were aspects of the training in the included studies. This is important to facilitate healthcare professionals engaging more positively with patients to develop strong therapeutic relationships and shared understandings, which encourages patients to take agency in their own care. Developing a shared understanding in the included studies was aided by the provision of plausible explanations. Healthcare practitioners reported that a strong learning point was being able to make the link between interrelated factors namely symptomatic presentation and psychosocial stressors. This facilitated patients to endorse an emotional response or attribute psychosocial distress to their symptoms.

Whilst psychosocial stressors can provide an explanation for environmental or interpersonal factors that can enable the development, perpetuation or exacerbation of symptoms, a gap within existing training programmes was the lack of explanation around psychosocial and physiological interrelating factors. There is a strong association between traumatic experiences and health outcomes due to the physiological changes trauma induces on the body [36]. Studies have shown that prevalence rates of PPS are much greater when an individual has experienced child and or adult trauma, leading them to be three times more likely to develop PPS [37–40]. Kendal-Tackett [41] refers to the importance of mind–body links, which describe how adverse experiences are held within the body and present as ill health when not appropriately dealt with.

The use of mind–body explanations in conjunction with a positive PPS diagnosis offers patients valid and plausible justification for their symptoms, and therefore should be incorporated into undergraduate teaching and post-qualification continuous professional development (CPD). A curriculum review is required due to PPS being completely absent from some curricula in UK medical schools, and where it is taught it accounts for just one day of teaching, typically during psychiatry placements [42]. Furthermore, Yon et al. [43] reported that just 11% of newly qualified doctors received formal PPS teaching on their programmes. Not only does this risk PPS reinforced as a product of mental health difficulties but that PPS patients are illegitimate users of medical services. This is reflected in the attitudes of some experienced medical professionals who may consider PPS as less severe than physical symptoms with identifiable pathology; attitudes potentially acquired by medical students [34].

We reinforce Yon and colleagues’ [43] notion that a rigorous and systematic approach to formally implement PPS into medical education is urgently required, but this will not address the low uptake of training by experienced medical professionals. Salmon et al. [35] attribute this to doctors devaluing their psychological skills, which existing training programmes are fundamentally built up on. Breaking the mind–body dualism ideology of PPS is an important consideration for educators when designing training packages, and they must ensure that training is clearly intended to develop psychologically informed practice to work with patients holistically at the interface of somatic and psychological care [44]. We recommend educators utilise the biopsychosocial model, as endorsed by several of the included studies [21, 22, 33], which offers a theoretical framework that would allow educators to develop training programmes that considers the multifaceted presentation of PPS.

Despite the present review identifying that healthcare practitioners were more confident to query psychosocial factors after engaging in training, they did not explore further when disclosures were made. Given the complexity and challenges of managing PPS already, it is likely that healthcare practitioners wanted to avoid ‘opening a can of worms’ as they did not have any direction where to go next. A gap across the training programmes is that healthcare practitioners were not encouraged to work with colleagues across services or specialities. NHS England [45] enunciates that collaborative practice should be adopted by all healthcare professionals, creating multidisciplinary working across organisational boundaries to ensure patients receive support that is effective and efficient to meet patient’s individual needs [46].

### Strengths and Limitations

Not all positive results showed statistical significance, however, improvements should be acknowledged as these demonstrate clinical significance. Sharma [47] asserts that when study outcomes are interpreted to determine the effectiveness or efficacy of an intervention, we can look beyond the *P value* threshold. Clinically relevant factors refer to improvements in the provision of patient care, leading to improvements in quality of life, individual physical functioning, mental health, general wellbeing, and the mitigation of physical symptoms [47]. One drawback of the present review is that whilst interventions aimed to improve quality of care for patients who present with PPS, not all studies capture patient outcomes. However, where they have, improvements in physical functioning, reduced symptom severity, psychological wellbeing and reduced healthcare utilisation were reported.

A second limitation is the heterogeneity of intervention designs and outcome variables across the included studies, which made the synthesis of the data challenging. The variation in intervention frameworks and lack of unifying theories limits the generalisability of the results to the wider population [48, 49]. Whilst the theoretical underpinnings and intervention frameworks differed among studies, the learning objectives and techniques were similar across interventions i.e., all interventions incorporated educational elements and experiential learning including skills development through role play. The variation of outcome measures gave insight to multiple factors that affect both patients and healthcare practitioners, addressing fundamental topics that are highly relevant in the assessment, treatment and management of patients who present with PPS.

### Implication for practice and future research

Some very clear learning points for developing future training programmes were identified. Firstly, the blended learning approach was reported to be useful, particularly the use of educational e-learning elements and in-person workshops focusing on skills development. However, online modules should be short and concise and face-to-face workshops should focus predominantly on skills development/practice via role play particularly when explaining explanatory models around symptomatic presentation. Given the prevalence of traumatic experiences among patients who present with PPS diagnoses, integrating mind–body explanations into healthcare practitioners’ clinical practice would be beneficial to providing valid and plausible explanations for symptoms.

Where possible, healthcare practitioners would also benefit from the opportunity to discuss patient cases for further advice and guidance during face-to-face sessions. It should be acknowledged that current time constraints and pressures on health services may prevent the development and implementation of a comprehensive PPS training programme. However, improving medical professionals’ interpersonal skills has been universally recognised [50], therefore where possible, we would recommend that PPS knowledge including mind–body explanations are embedded into existing communication programmes.

Only one study included a tool that facilitated a structured consultation [33], however, others reported that guidance on structuring consultations would be useful. Moving away from the biomedical model of healthcare to a biopsychosocial model would be useful to guide holistic consultations. In terms of structuring the consultation, identifying tools that facilitate brief discussions around each domain i.e., the biological/physiological, the psychological and the social will enable healthcare practitioners to have more structured, focused, and holistic conversations to identify each patient’s individual needs. The model can then be used to support identifying management techniques, including encouraging patients to take agency in their own care. To encourage multidisciplinary team working and collaborative practice, training should involve raising awareness of other services that healthcare practitioners can signpost patients to e.g., Social Prescribers for support around housing, finances, etc., or Health Coaches for support to improve health behaviours.

Finally, studies that took place in primary and secondary care were included in the present review due to the prevalence of PPS across both settings. By including studies from each mode of healthcare, this enabled us to identify any potential differences in the effectiveness of interventions between primary and secondary care settings. However, of all the included studies, just one study took place in secondary care where the study aims and objectives and method of delivery were like those that took place in primary care. Therefore, it was not possible to tease out differences of intervention effectiveness or techniques utilised between both settings. Additional investigations in secondary care are required to obtain further insight into the operationalisation of clinical skills interventions when working with PPS and to identify any training needs that may differ to those identify in the present review.

## Conclusions

This review provided a synthesis of existing evidence to support the development of healthcare practitioners’ clinical skills to improve PPS consultations. Due to the few included studies using a wide variety of outcome measures, we cannot firmly conclude that improved healthcare practitioner and patient outcomes would apply to the wider population in this field. Our findings, however, do endorse the importance of developing healthcare practitioners’ skills to look beyond patients’ symptomatic presentation and assess, treat, and manage them holistically. The review highlights the practical application of skills that can be utilised in daily clinical practice to better support patients, including the importance of patient self-management of symptoms. Future training should seek to widen the application of explanatory models to include physiological and psychosocial interrelated factors to explain symptoms and encourage multidisciplinary team working across organisational boundaries. Healthcare providers and educators should endeavour to formally implement PPS knowledge and skills development into undergraduate teaching, newly qualified post-graduate training and experienced medical professionals’ CPD.

## Data Availability

All data generated or analysed during this study are included in the published article.

## Appendix

**Table 4:** Full search strategy

Host	Database	Search No	Search Term
EBSCO	AMED MEDLINE P&BSC PsychInfo CINAHL	S1	“persistent physical symptoms” OR “medically unexplained symptoms” OR “functional somatic symptoms”
		S2	“training” OR “learning” OR “development”
		S3	“service delivery” OR “healthcare provision” OR “quality of care” OR “clinical skills” OR “physician–patient relationship”
		S4	S1 AND S2 AND S3
Ovid	EMBASE	S1	(persistent physical symptoms or medically unexplained symptoms or functional somatic symptoms).ab
		S2	(training or development or learning).af
		S3	(service delivery or healthcare provision or quality of care or clinical skills or physician–patient relationship).af
		S4	S1 AND S2 AND S3
-	Scopus	S1	TITLE-ABS-KEY (persistent physical symptoms) OR TITLE-ABS-KEY (medically unexplained symptoms) OR TITLE-ABS-KEY (functional somatic symptoms)
		S2	TITLE-ABS-KEY (training) OR TITLE-ABS-KEY (development) OR TITLE-ABS-KEY (learning)
		S3	TITLE-ABS-KEY (service delivery) OR TITLE-ABS-KEY (healthcare provision) OR TITLE-ABS-KEY (quality of care) OR TITLE-ABS-KEY (clinical skills) OR TITLE-ABS-KEY (physician–patient relationship)
		S4	S1 AND S2 AND S3
ProQuest	Nursing and Allied Health Source	S1	ab(persistent physical symptoms) OR ab(medically unexplained symptoms) OR ab(functional somatic symptoms
		S2	training OR development OR learning
		S3	(service delivery) OR (healthcare provision) OR (quality of care) AND (clinical skills) AND (physician–patient relationship)
		S4	S1 AND S2 AND S3

**Table 5:** Summary of the included studies results

	**Study**	**Intervention and design**	**Outcome summary**	**Notes for intervention development**
1	Abrahamsen et al. (2022) [29]	The Individual Challenge Inventory Tool (ICIT) Qualitative	Themes: 1) The ICIT to facilitate structured consultations: helped to sort out, clarify, substantiate, and concretise patients’ issues 2) HCPs perception of the patient’s experience: activity plan helped patients achieve a sense of control; an important self-help tool; gave HCPs something specific to offer patients 3) The ICIT as a tool to scope assessment of sick leave and treat patient, and encourages patients to reflect on positives, rather than limitations 4) Short-comings and challenges using the ICIT: HCPs identified importance of activity plan; demand characteristics of patients to meet HCPs expectations; one patient – ICIT trivialised symptoms; ICIT useful to refine patient’s challenges, in addition to reflect some responsibility back to the patient	Structured consultations Patient sense of control Self-help tool / something to offer patients Draw on strengths, not focus on limitations Patients to take agency
2	Frostholm et al. (2005) [21]	The Extended Reattribution Model (TERM) RCT	Primary outcomes • Patient satisfaction higher when consulting trained HCP - Particularly when feeling uncertain about their health problem Patient predictors (higher chance of dissatisfied patients) • High illness worries • High symptomology • Higher levels of emotional distress • Patients’ illness perceptions (negative emotional representation, high levels of uncertainty, perceived negative consequences, long timeline perspective) • Psychosocial issues (feeling down, worried, state of mind, problems in family, personality)	Important to think about patients holistically i.e., psychological/emotional distress Psychosocial issues should be embedded within consultations
3	Houwen et al. (2022) [30]	Intervention Mapping Framework Mixed methods	Self-efficacy (assessed at three time points) • Significant increase in scores across all time points Qualitative feedback 1) Benefit of training programme: e-learning good theoretical practice for in-person training days; and education that integrates e-learning and face-to-face learning needs 2) Acquisition of skills: learning to conduct thorough exploration of patient’s symptoms; more aware of attitudes and language used, importance of shared understanding, introduced to several explanatory models, improve referral letters 3) Recommendations for training adaptations: HCPs still experience difficulties providing plausible explanations – more time practicing this in face-to-face sessions; e-learning extensive and time consuming (max 420 min / 7 h)	Blended learning useful to incorporate theory (online) and skills practice (in-person) In-person to focus on explaining explanatory models and creating a shared understanding of symptoms Short and concise pre-workshop modules
4	Morriss et al. (2006) [31]	The Extended Reattribution Model (TERM) Quantitative	One single feasibility questionnaire - 33% training very useful in their job - 44% training useful in their job - 15% unsure of how useful the training was - 8% training had very little use - 82% felt confident or very confident in managing patients with PPS after training - 18% uncertain or unchanged in confidence - 44% training methods helpful - 48% training methods very helpful (except one specific aspect of course -not specified in results) - 8% training methods unhelpful - 18% longer course to practice role play - 18% chance to discuss difficult cases and techniques New learning achievements - 48% better or alternative ways of making the link between interrelated factors - 22% provision of structure to consultations - 18% more confident to openly discuss PPS with patients	Making the link between interrelated factors Focus on explaining explanatory models Identifying a structure to consultations HCPs would benefit from the opportunity to discuss difficult cases
5	Morriss et al. (2007) [32]	The Extended Reattribution Model (TERM) RCT	Substantial improvements were shown in the training group in terms of doctor-patient communication consistent with the TERM model. HCPs explored factors such as health beliefs, making the link explanations, and feeling understood chatter more than the controls Secondary outcomes – TERM associated with - Non-significant improved patient satisfaction - Higher proportion of patients felt they knew the cause of their symptoms; endorsed an emotional cause - Worse self-rating of overall health, higher cases of anxiety, beliefs health issues may last longer, health consequences will be more serious and less under the patient’s control - No effects on psychological wellbeing (depression, health anxiety) or use of healthcare resources	Making the link between interrelated factors to explain symptoms
6	Morriss et al. (2010) [20]	The Extended Reattribution Model (TERM) RCT	Psychosocial chatter after TERM training: - Substantial increase in GP prompt for psychosocial information concerning symptoms - Increase in patients prompting psychosocial information - Did not increase GP to explore psychosocial disclosures further - Increased GPs provision of psychosocial explanation - Decreased GP advocation for somatic intervention - GPs speech: average of 6 utterances concerning the appropriateness of somatic intervention and an average of 2 utterances of psychosocial explanation - Increased patients’ disclosure of psychosocial problems - Approx. 50% patients disclosed a new psychosocial problem - 25% patients in IG: 2 or more utterances of psychosocial disclosures, 10 or more elaborating on psychosocial disclosure, 4 or more utterances of psychosocial explanation for their PPS	Psychosocial stressors an important consideration in consultations – i.e., holistic assessment
7	Schaefert et al. (2013) [33]	Special Collaborative Group Intervention (speciAL) RCT	Primary outcome: SF-36 (PSC at 12 months) - Non-significant improvements in both groups; great improvements in IG (but very little) - Physical functioning significant at 12 months - Somatic symptom severity improved at 6 months (significant), lasted to 12 months but no longer significant Secondary outcomes: SF-36 (MCS) - Significant improvements in both groups; larger improvements in IG (55% IG versus 34% CG) - Improved by 4-points or more on MCS (used as threshold to determine clinical change for PPS in primary care - At 12 months: vitality and emotional functioning significant Less psychosocial distress, less health anxiety Healthcare resource utilisation: Number of visits to GP or medical specialists decreased in both groups, significant in IG group; use of antidepressants lower in IG group compared to CG – declined over time in both groups but only reach significance in IG at 6 months)	Aim of the intervention was to improve patient coping with persistent physical symptoms – understanding and accepting symptoms likely explain areas of improvement
8	Weiland et al. (2015) [22]	An evidence-based communication programme using techniques from CBT RCT	Trained HCPs showed (in comparison to CG): - Larger increase in exploring patient’s cognitions, impact of symptoms on behaviour, environment, and emotions - Worked in a more person-centred way, explained interrelating factors more frequently - No effects for making plans and follow-up appointments - Better interviewing and information-giving skills in PPS consultations HCP feedback: • Training programme useful for daily practice • Scored 2.79 on a 3-poing Likert scale • Exercise skills, literature and during of training reported as useful • Despite useful feedback, HCPs reported consultations with patients from different ethnic backgrounds as extremely difficult (factors: time, professional interpreters, knowledge of cultural diversity)	Engage patients holistically Working in a person-centred way
